# Factors Behind Junk DNA in Bacteria

**DOI:** 10.3390/genes3040634

**Published:** 2012-10-12

**Authors:** Rosario Gil, Amparo Latorre

**Affiliations:** 1 Institut Cavanilles de Biodiversitat i Biologia Evolutiva, Universitat de València, Apartado Postal 22085, 46071 València, Spain; Departament de Genètica, Universitat de València, Dr. Moliner, 50, 46100 Burjassot (València), Spain; 2 Área de Genómica y Salud, Centro Superior de Investigación en Salud Pública (CSISP), Avenida de Cataluña 21, 46020 Valencia, Spain; E-Mail: amparo.latorre@uv.es

**Keywords:** junk DNA, pseudogenes, intergenic regions (IGR), insertion sequences (IS), genome degradation

## Abstract

Although bacterial genomes have been traditionally viewed as being very compact, with relatively low amounts of repetitive and non-coding DNA, this view has dramatically changed in recent years. The increase of available complete bacterial genomes has revealed that many species present abundant repetitive DNA (*i.e.*, insertion sequences, prophages or paralogous genes) and that many of these sequences are not functional but can have evolutionary consequences as concerns the adaptation to specialized host-related ecological niches. Comparative genomics analyses with close relatives that live in non-specialized environments reveal the nature and fate of this bacterial junk DNA. In addition, the number of insertion sequences and pseudogenes, as well as the size of the intergenic regions, can be used as markers of the evolutionary stage of a genome.

## 1. Introduction

### 1.1. Genome Size and Junk DNA

The availability of an increasing number of complete genome sequences has allowed researchers to extract general rules about their shape and dynamics, as well as to analyze the molecular and evolutionary forces involved. Eukaryotes have a wide range of genome sizes, from the 6.5 Mb of the pneumonia-causing fungi *Pneumocystis carinii* f. sp. *muris* to the 133 Gb of the marbled lungfish *Protopterus aethiopicus* [1]. However, it has been demonstrated that theincrease of genome size is not correlated with an increase in complexity or gene number, as bigger eukaryotic genomes tend to increase the amount of non-coding DNA (ncDNA) and contain large amounts of transposable elements [2]. The high percentage of ncDNA present in higher eukaryotes genomes was already noticed in the 1970s, when it was considered as “junk DNA” [3]. However, it has become obvious that many of these non-coding sequences are fully functional. Therefore, it would be more appropriate to refer to junk DNA only when we are talking about DNA that has “little obvious function” and “contributes little or nothing to the fitness of the organism” [4]. This would include inter- or intragenic regions that have no known function, pseudogenes, repeated sequences and selfish DNA such as transposons and viral elements.

On the other side, prokaryotic genomes are in general smaller and less variable than eukaryotic ones, ranging from less than 200 kb to more than 11 Mb (http://www.genomesonline.org). The size distribution in the different bacterial taxonomic groups shows that species with large and small genomes coexist in the different lineages and important size variation can even be observed among strains of the same species. *Escherichia coli* genomes can range from 4.6 to 5.7 Mb; *Procholoroccus marinus*, from 1.6 to 2.7 Mb; *Pseudomonas fluorescens*, from 6.3 to 7.1 Mb, or *Buchnera aphidicola,* from 0.4 to 0.7 Mb. All these examples illustrate that genome size is highly variable in prokaryotes and that, contrary to eukaryotes, it may change drastically even within a short divergence time. Variations in genome size are mostly related to the bacteria lifestyle, although different mechanisms seem to be acting in free-living and host-dependent bacteria [5]. Regarding gene content, and also contrary to eukaryotes, bacteria and archaea genomes tend to be very compact, with genes occupying around 90% of the genome for most of the species [6]. Intergenic regions (IGRs) are usually short but variable in size, with mean values as small as 3 bp in *Pelagibacter ubique*, 85 bp in *E. coli*, or 151 bp in *Yersinia pestis* [7]. Therefore, in general, genome size shows a strong positive correlation with gene number, although exceptions to this compactness are accumulating in recent years. Many sequenced bacterial genomes present a lower gene density than average due to the accumulation of different types of sequences that, similarly to eukaryotic genomes, can be considered “junk DNA”. Some organisms possess a large quantity of pseudogenes, as it is the case of *Mycobacterium leprae* [8], *Serratia symbiotica* SAp [9] or *Sodalis glossinidius* [10,11], while others, such as *S. symbiotica* SCs [12] or *Rickettsia prowazekii* [13], show long IGRs. There are also genomes where genetic parasites, such as transposable elements and bacteriophages, are highly present, as it is the case of SOPE [14]. In this review we will not take into consideration bacteriophages as junk DNA because it has been shown that genes of a phage origin have been co-opted by different bacteria for their own profit. Thus, bacteriophages are known to carry key virulence factors for pathogenic bacteria [15] and, although their roles in symbiotic bacteria are less understood, Oliver *et al.* (2009) [16] suggested that a phage-encoded toxin is responsible for the protecting role of *Hamiltonella defensa*, a facultative symbiont of the pea aphid *Acyrtosiphon pisum,* against the attack of parasitoids. Furthermore, though several studies are trying to manage this problem [17,18,19], the present standards of annotation for genomes available in the databases make difficult to assess when a putative functional gene has a phage origin or is just a non-functional remnant of an ancestral phage gene [20].

### 1.2. Bacterial Endosymbionts as a Model

Our group has been involved for more than a decade in the study of the genomes of endosymbionts, bacteria that live inside specialized eukaryotic cells. Many bacteria live in obligate association with eukaryotes (see [21] and [22] for complete reviews on the subject). Based on the effect of the bacterium on its host, these relationships are classified as mutualistic, commensal or parasitic but, from the bacterial point of view, the basic requirements to successfully maintain this kind of association are the same. They need to overcome the physical, cellular and molecular barriers presented by the host to achieve survival, proliferation and the infection of a new host. Therefore, the evolutionary processes suffered by both pathogens and mutualists are very similar. The rich and stable environment provided by the host cells leads to dramatic changes in the bacterial genome composition due to a relaxation of the selective forces on genes that become non-essential in the new environment, alongside an increase in the effects of genetic drift caused by bottlenecking over bacterial generations. These changes include a gradual reduction in genome size as well as variation in the coding capacity and gene density, together with changes in the number of genes and pseudogenes, and in the presence of insertion sequences (IS). Although endosymbiotic bacteria that keep a long term relationship with their hosts have extremely reduced and (usually) compact genomes, without traces of phage remnants, sequences acquired through horizontal gene transfer (HGT) and ISs and with limited amounts of pseudogenes, newly acquired endosymbionts still retain many of these features in their genomes, probably reflecting an intermediate step before the massive genome reduction starts. To better understand the molecular and evolutionary bases of endosymbiosis, the genomes of bacteria in different stages of the symbiotic integration process have been analyzed. These genomes and their comparison with those from their free-living relatives can be also useful in understanding the fate of bacterial junk-DNA. Or, on the other hand, the analysis of junk DNA can be used as a marker of the integration process of endosymbiotic bacteria.

## 2. The Impact of Pseudogenes in Bacterial Genomes

Pseudogenes are inactivated copies of known genes, which present an erosion or disruption either of their reading frames or their regulatory regions. They are usually detected by comparative analysis with their functional orthologs in close relative species. The alignment of such orthologous genes allows the detection of mutations, insertions/deletions (indels), frameshifts, premature stop codons and insertion of transposable elements, all of which cause the appearing of a truncated protein, or the inactivation or loss of essential functional domains. However, it is not always easy to assess the functional status of each annotated coding region within a genome for several reasons. First of all, orthology cannot be detected above a threshold of divergence between sequences. Second, not all frameshifts, indels, stop mutations and rearrangements will inactivate a gene and there is a wide length variation of homologous proteins within families. An additional problem is that there is no clear definition of what has to be considered a pseudogene and there are inconsistencies in the methods used in different studies, so that it is not always possible to compare the pseudogene content from different genomes. In some studies, any shortened open reading frame (ORF) is annotated as a pseudogene [23], while in others considerably shortened homologs are annotated as genes if they keep complete domains specifying some function [24]. Lerat and Ochman (2005) [25] considered a pseudogene only when more of 20% of the length of the primary sequence of the encoded protein was lost, while some other studies excluded from this category impairments in homology matches within a “cutoff” region at either end, considering that slightly shorter alignments can reflect functional protein [26]. The most widely used method to identify non-functional genes involves the comparison of nucleotide substitution rates at synonymous (*K*_s_) and non-synonymous sites (*K*_a_), using the *K*_a_/*K*_s_ test [27]. Regions without functional constraints, such as pseudogenes, are expected to have *K*_a_/*K*_s_ ratios not significantly different from one. However, comparative analyses can only detect pseudogenes in genes that have orthologs in other available genomes, and only if the inactivation is due to truncations or disruptions of the original ORF, which represents only a fraction of the potentially inactivated genes. Genes that have been inactivated by missense mutations or changes in regulatory regions will also remain undetected by these methods. 

At the beginning of the Genomics era, pseudogenes were thought to be unusual in bacteria. This idea has completely changed now that it is possible to perform comparative genomics on different strains of the same species, on closely related species with different lifestyles or on those living in different environments. In fact, when the *E. coli* MG1665 genome was first reported, only one pseudogene was annotated among its 4288 coding regions, but recent studies revealed that this genome contains about 100 genes that retain less than 20% of the annotated sequence in other *E. coli* strains, some of which (at least) are likely to be non-functional [28]. Nevertheless, pseudogenes were early noticed in significant amounts in some bacterial pathogens such as *R. prowazekii* [13] or *M. leprae* [8], the latter being one of the most extreme cases known to date, with 1614 protein-coding genes and 1133 annotated pseudogenes, 40% of its 3.2-Mb genome. Since then, several comprehensive studies have been performed trying to extract general rules that explain the dynamics of pseudogenes within bacterial genomes. 

Liu *et al.* (2004) [26] analyzed 64 prokaryote genomes and defined a conservative set of conditions to detect their proportion of pseudogenes. In this analysis, they do not include hypothetical or putative proteins, because a large proportion of them could be over-annotated. However, in order to maximize the efficiency of pseudogene finding, they searched for remnants of ancient genes in the regions that had been considered as IGRs in the original annotations. Using this approach, they found that pseudogenes are pervasive in prokaryotes, accounting for 1 to 5% of most analyzed genomes. They didn’t find a clear correlation between percentage of pseudogenes and lifestyle: taking aside the extreme case of *M. leprae*, the pseudogene fraction of the analyzed archaea, non-pathogenic and pathogenic bacteria were fairly similar (3.6, 3.9 and 3.3% respectively). However, and as mentioned in the introduction, it has been shown later on that some bacteria that have recently adopted an obligate host-dependent lifestyle (either mutualistic or parasitic) present higher amounts of pseudogenes, probably related with a rapid change to the new environments in which some previously encoded functions are no longer needed. The rapid adaptation to new environments also explains the situation of *M. leprae* and its differences with its close relative *M. tuberculosis* [29] in that, with 3959 protein-coding genes, has only six identified pseudogenes. A comparative analysis of both genomes suggested that the pseudogenes in *M. leprae* have degenerated by gene-by-gene inactivation mostly after the divergence of these two clades [30]. Additionally, genomes from host-dependent symbionts that are suffering a genome reductive syndrome are known to contain many pseudogenes in regions that were previously annotated as IGRs. Thus, when the genome of *R. prowazekii*, the a-proteobacteria that causes typhus was sequenced [13], it was found that a considerable portion of its 1.1-Mb genome (22.9%) is covered by ncDNA and pseudogenes. A comparative analysis of this genome with the genome of three representative species of the genus *Rickettsia* showed that most of the IGRs were, in fact, remnants of ancestral genes with a high degree of degradation [24,31]. Even in smaller genomes, such as those from four different strains of *B. aphidicola* (the bacterial endosymbiont of aphids), longer IGRs in one strain contain the remnants of lost genes in other lineages [32]. 

As it would be expected, the pseudogenes identified by Liu *et al.* (2004) [26] cluster mainly in specific families related with environmental responses (such as specific nutrient transporters or processing, and related with antigenic variation). Other disabled genes correspond to hypothetical and unknown proteins, belong to transposable elements and bacteriophages, or are remnants of horizontally-transferred genes. Later on, Lerat and Ochman (2005) [25] determined the pseudogene content of 11 available complete genomes from four bacterial genera (*Staphylococcus, Streptococcus, Yersinia* and *Vibrio*), each of which include at least one human pathogen, which were supposed to accumulate pseudogenes compared with their free-living relatives. In addition to the gene families identified in the previous study [26], in obligate host-associated bacteria several broadly distributed genes involved in nucleotide processing, repair or replication appear as pseudogenes. The loss of genes needed for DNA repair and recombination are also among the first losses detected in bacteria that have recently acquired an obligate endosymbiotic lifestyle [33], thus contributing to the accumulation of pseudogenes in these species [34]. 

## 3. IS Elements Shaping Bacterial Genomes

Mobile genetic elements, such as phages, plasmids and transposable elements are widespread in prokaryotes, where they can represent a significant proportion of their DNA and play important roles in shaping their genomes. The simplest and most abundant transposable elements in bacteria are ISs. Although several classification schemes have been proposed, the one defined by Mahillon and Chandler (1998) [35] has become the most widely used. It is the format used by ISfinder (www-is.biotoul.fr) [36], a repository of ISs isolated from bacteria and archaea that also provides extensive background information, as well as an updated and comprehensive classification in IS families and subfamilies, and a proposal for a coherent nomenclature. These small elements are very compact and consist on a short DNA sequence, usually between 0.6 and 2.5 kb in length, able to translocate within and among replicons. A typical IS element only codes for proteins involved in its transposition, flanked by short inverted repeats (IR) of around 10 to 40 bp. Usually, their transposition generates a small duplication (2 to 14 bp) of the target DNA flanking the insertion point. Based on similarities in their transposases and IRs, and the length of their target site sequence, ISs can be grouped into 20 major families. IS elements are important factors involved in genetic variability, since they can promote genomic rearrangements, and generate mutations by their insertion within genes or regulatory sequences. This capacity to generate genome damage is probably one of the reasons why transposition is usually strongly regulated and maintained at a low level in free-living bacteria [37]. In fact, most bacterial genomes contain only a few copies of a limited number of IS types [38]. However, in bacteria that have recently adopted an intracellular lifestyle, there is a reduction in the selective pressure to keep the ISs regulated, and a massive expansion of those elements can take place (see below). 

As it happens with the pseudogenes, the quality of IS annotation in sequenced genomes is highly heterogeneous due to the different annotation methods and nomenclature used by different researchers, and the diverse interests that drive the functional analysis of a given genome. In fact, it is not uncommon that annotation focuses only on the potential protein-coding sequences included in the elements, but ignores their IR boundaries and the directed repeats generated by their insertions, as well as the vestiges of ancestral ISs [36]. Nevertheless, several global surveys of available bacterial genomes have been performed in recent years, allowing conclusions to be obtained regarding the distribution, dynamics and evolution of these elements.

Many different hypothesis have been proposed to explain the abundance and variability of IS elements among prokaryote genomes. Wagner *et al.* (2007) [38] used IScan, a free open source package, to examine the more than 2000 IS elements present in 438 completely sequenced bacterial genomes in the most comprehensive analysis to date. They found a high homogeneity pattern of IS families across vast taxonomic scales, consistent with previous and more limited works [39,40,41]. Such high-sequence homogeneity could be explained by the rapid spreading of ISs within a genome, in addition to other genetic mechanisms such as gene conversion. In the evolutionary scenario proposed by Wagner *et al.*, after an IS enters a genome, its copy number expands rapidly through transposition. Consequently, there is a low degree of diversity among the different copies of an IS present in a given genome. Eventually, due to the deleterious effect of their accumulation, IS elements tend to disappear and may become extinct from the lineage. However, later on, it may be reintroduced through HGT. The impact of HGT in the spreading of these sequences is revealed by the presence of closely related IS elements of the same family in non-closely-related genomes. However, HGT appears to be necessary but not sufficient for the presence of ISs, since their abundance within a genome does not depend on the level at which genomes are invaded by the elements [42].

Touchon and Rocha (2007) [42] performed a comprehensive reannotation and analysis of the putative functional ISs present in 262 prokaryote genomes in order to test for IS family specificity, the influence of host genome size, pathogenicity, or human association in the IS abundance or density. They found that IS distribution in prokaryotic genomes strongly correlates only with genome size, probably due to a decrease on the density of highly deleterious insertion sites with genome size. They hypothesized that IS abundance is mostly determined by selection and, therefore, the effective population sizes of the microorganism would strongly influence IS abundance. Thus, the high increase in the amount of ISs that has been detected in some bacterial pathogens, even though they have smaller genomes, would be the consequence of a recent reduction of effective population sizes, not of their parasitic lifestyle. However, it must be taken into account that it is their host-dependent lifestyle that determines that many genes become redundant or superfluous, which also diminishes the effectiveness of natural selection, because there are more sites that can be substrate of IS transposition without affecting the fitness of the microorganism.

On a more recent study, Newton and Bordenstein (2011) [43] analyzed 384 bacterial genomes on the light of their phylogenetic relationships, genome sizes and ecology, in order to test whether there is a correlation between any of these factors and the mobile elements density (which in this study included phages, plasmids and transposable elements). They found that the density of mobile DNA elements only correlates with bacterial ecology. ISs account for the majority of mobile DNA elements in nearly half of the analyzed genomes, and there is a significant variation on the amount of them depending on the bacterial lifestyles (free-living, facultative intracellular and obligate intracellular bacteria), and also between horizontally and vertically transmitted obligate intracellular bacteria. The increase in the amount of IS elements in bacteria that have recently evolved as specialized pathogens or acquired an intracellular lifestyle had already been noticed in previous studies [44]. Since these bacteria sequestered inside eukaryotic cells can not exchange material with other bacteria through HGT, the massive presence of ISs must be due to an increase in the replicative transposition of elements that were resident at the onset of the obligate symbiosis [45], when many genes that had become non-essential can accumulate ISs without a detrimental effect. In addition, the high abundance of IS elements is also an important source of chromosomal rearrangements suffered by these genomes at this point [46]. Some studies emphasize that the persistence of IS elements in bacterial genomes can not be only explained by the balance of their propagation as selfish elements and the control of the host genome to avoid deleterious effects, but also due to their ability to promote adaptive evolution of the host genomes by generating beneficial mutations that increase the fitness of the host [47]. However, such benefits do not seem to be acting on extremely reduced genomes with a large evolutionary history with their hosts, since IS elements have been completely lost in their genomes. Their absence in the latter stages of the endosymbiosis can mostly explain the extreme chromosomal stasis observed in the genomes of the different *B. aphidicola* strains that have been sequenced [48].

## 4. Junk DNA as a Marker of the Symbiotic Integration Process

In recent years, new information has been obtained by the study of bacterial endosymbionts in different stages of their relationship with their respective hosts. Several cases are especially useful to illustrate the dynamics of junk DNA in the genomes of bacteria that acquire a specialized lifestyle and will be analyzed in detail in this section and summarized in Table 1. 

### 4.1. Serratia symbiotica, the Missing Link from Free-Living to Obligate Mutualism

*S. symbiotica* is a g-proteobacterium that appears in symbiotic association with different aphid species. In most cases, as for the pea aphid *A. pisum,* it appears as a facultative symbiont (strain SAp) [9]. However, in the case of the cedar aphid *Cinara cedri*, *S. symbiotica* SCc has established a permanent and obligate consortium with *B. aphidicola* [12], the primary endosymbiont of aphids. Therefore, S*. symbiotica* SCc is present in all cedar aphid populations and both bacteria maintain a metabolic complementation for the provision of some essential nutrients, such as tryptophan, as revealed by functional analyses of their genomes. It is worth noticing that *B. aphidicola* BCc from *C. cedri* possess the smallest genome among all the sequenced *Buchnera* strains [52].

**Table 1:** Relevant genomic features of selected bacteria with different lifestyles

Species	Lifestyle	Genome Size (kb)	CDS	Pseudogenes	IGR Mean Size (bp)	% Coding Density	Presence of ISs (%)	Data Source
*M. leprae* TN	human parasite	3,268	1614	1293	ND	ND	Yes	[30,49]
*M. tuberculosis* H37Rv	human parasite	4,411	4006	6*	ND	ND	1.5	[29,44,50]
*S. symbiotica* SAp	pea aphid facultative symbiont	2,789	2098	550	204.3	60.9	Yes	[9,12]
*S. symbiotica* SCc	cedar aphid facultative symbiont	1,763	672	58	1672.01	38.7	No	[12]
*S. proteamaculans*	free-living	5,496	4942	12	165.67	87.1	Yes	[12]
*B. aphidicola* BAp	pea aphid obligate endosymbiont	656	574	1	126.9	86.7	No	[12,51]
*B. aphidicola* BCc	cedar aphid obligate endosymbiont	422	362	3	135.8	90.0	No	[52]
*S. glossinidius* str. "morsitans"	tse-tse fly facultative symbiont	4,293	2516	1501	ND	50.9	2.72	[10,11]
*W. pipientis w*Mel	*D. melanogaster* reproductive parasite	1,268	1270	94	ND	80	7.7	[44,53]
*W. pipientis w*Ri	*D. simulans* reproductive parasite	1,445	1150	114	ND	ND	10	[54]
*W. pipientis w*Pip	*C. pipiens* reproductive parasite	1,482	1386	97	ND	82	Yes	[55]
*W. pipientis w*Bm	*B. malayi* obligate endosymbiont	1,080	806	98	ND	67	5.4**	[56]
*R. prowazekii* str. Madrid E	human parasite	1,112	835	12	ND	76	0.3	[13]

* Excluding IS elements. ** Various repeats including ISs. ND: non-determined

The genome comparison of both obligate and facultative *S. symbiotica* strains and other free-living *Serratia* reveals the gradual changes in gene content at different stages in the transition from free-living to endosymbiosis. *S. symbiotica* SCc has a moderately reduced genome (36.8%), compared to *S. symbiotica* SAp, and a 67.7% reduction compared to free-living *Serratia* such as *S. proteamaculans*. However, the data indicating genome decay are more extreme when the coding capacity is compared: the obligate strain SCc presents only 672 protein-coding genes and 58 pseudogenes, whereas the facultative strain SAp has 2098 genes and 550 pseudogenes. Thus, the overall coding density of strain SCc is 38.7%, the lowest among insect endosymbionts described so far. As a consequence, the IGRs are very long in this strain, with an average length of 1,672 bp, the highest among all endosymbionts analyzed. No traces of homology have been found in these IGRs compared to coding regions of other sequenced bacterial genomes. This ncDNA must represent ancient pseudogenes that are being gradually eroded until their total (or almost total) disappearance, as it has happened in ancient obligate bacterial endosymbionts [57]. Altogether, these data indicate that this genome is in the last steps of genomic degradation but previous to that of long-term endosymbionts such as *B. aphidicola*. In fact, if we substitute the size of the IGRs in *S. symbiotica* SCc for the size of these regions in *B. aphidicola* BCc (135.8 bp on average), the chromosomal length would be 771,075 bp, in the range of other obligate endosymbionts. In addition, no IS sequences remain in this strain, whereas they have been described in SAp as well as in free-living *Serratia*. An analysis of the genome synteny between both symbionts revealed that an important number of rearrangements have occurred in both *S. symbiotica* lineages: This fact is in accordance with the possibility of the presence of active mobile elements in their ancestor at the onset of the symbiosis, which are currently unidentifiable in the *S. symbiotica* SCc genome [12]. 

### 4.2. Wolbachia pipiensis, from Reproductive Parasite to Intracellular Mutualist

One of the cases most extensively studied correspond to different strains of *W. pipiensis*, an a-proteobacterium that has established parasitic and mutualistic symbiosis with different animal hosts [58]. Most *W. pipientis* strains are reproductive parasites of insects. The complete genome of the strains that parasite *Drosophila melanogaster* (*w*Mel) [53], *D. simulans* (*w*Ri) [54], and mosquitoes from the *Culex pipiens* group(*w*Pip) [55,59] are available. Additionally, *W. pipiensis w*Bm was identified as the obligate mutualistic symbiont of *Brugia malayi*, a human filarial parasitic nematode, and its genome was also sequenced [56]. All these bacteria have streamlined genomes compared with other free-living a-proteobacteria, as expected for host-associated microorganism, being more extreme in the case of the mutualistic strain *w*Bm. However, all of them also contain high levels of repetitive DNA and mobile elements. When the genome of the strain *w*Mel was determined [53], it was found that the mobile DNA identified didn’t have homologues in other a-proteobacteria but have been found in other *Wolbachia* strains. Therefore, the authors proposed that it was acquired short after the separation of the *Wolbachia* and *Rickettsia* lineages but before the radiation of the *Wolbachia* group. Another study revealed that most of the differences in genome size between *w*Mel with *w*Pip come from repetitive and mobile elements (not only ISs, but also prophages), and there is almost no overlapping between both genomes, which might be due to a recent invasion after the separation of both lineages or to different losses after such event [55]. Although many of the mobile elements seem to be defective, they must have played a key role in shaping the evolution of this genus, and are probably responsible for most of the genome rearrangements found among strains and compared with free-living relatives, similar to what has happened in *S. symbiotica* [12]. The comparison with *W. pipiensis w*Bm revealed that it also shares some repetitive elements with *w*Mel, although they are considerably less abundant in the mutualistic symbiont (5.4%) [56]. Additionally, the *w*Bm genome has an extremely low density of predicted functional genes, similar to what has been found in *R. prowazekii* [13] and *M. leprae* [8]. As in these other genomes, the main reason for such low-coding density is the presence of a considerable number of pseudogenes (which, even so, were underestimated at that time, since many putative ORFs correspond to fragmented former genes).

### 4.3. The Sodalis-like Group of Symbionts, at the Transition Point from Facultative to Obligate Symbionts

Phylogenetic analysis demonstrated the close relationship between *S. glossinidius*, secondary symbiont of the tse-tse fly and the primary endosymbiont of grain weevils of the genus *Sitophilus*, as it is the case of SOPE and SZPE, endosymbionts of rice and maize weevils, *S. oryzae* and *S. zeamays*, respectively. *Sitophilus* symbionts and *S. glossinidius* provide a good model for the study of the evolutionary transition from facultative to obligate-mutualism, because the divergence of these lineages from a common ancestor has produced two different symbiotic outcomes. The genome sequencing and reannotation of *S. glossinidius*, showed that about a third of it is composed of inactivated genes in different degrees of disintegration, and it also contains a limited amount of IS elements of five different types, representing 2,72% of the genome [10,11]. Among the 1501 identified pseudogenes, only 18 were originated by the insertion of an IS element. This big amount of pesudogenes confirms the very recent symbiotic association with their insect host [60,61], while it is clear that ISs have not been determinant in the degeneration of the genome. 

Although the genome sequence of SOPE is not yet available, preliminary studies showed that this genome has been massively invaded by IS elements, which have been estimated to represent more than 20% of the genome [14]. This is the most extreme case of IS abundance in any known bacterial genome. The presence of a Sodalis-like g-proteobacteria in cereal weevil lineages has been explained by a replacement of the ancestral endosymbiont *Nardonella* (present in the rest of the members of the family to which the rice weevil belongs) less than 25 million years ago [62,63], thus being a very young obligate mutualist. Therefore, it seems that after the establishment of the obligate symbiosis, the evolutionary path taken by SOPE differs from the observed in *S. glossinidius*, with a massive proliferation of at least four types of IS elements. Only two of these elements are shared with *S. glossinidius,* which as in the case of *Wolbachia*, indicates that they were present before the split of the two lineages, and the other two must have either been acquired by HGT in the ancestor of SOPE, or lost in *S. glossinidius*. The importance of the ISs in the process of pseudogenization can not be completely estimated until the full genome is available. Although it is known that many genes are interrupted by ISs, it is first necessary to determine if the interruption occurred on genes that were already pseudogenized in the common ancestor of *Sodalis* and SOPE, which seems to be the case. Preliminary studies performed in our group indicate that many genes inactivated by IS insertions encode hypothetical proteins or proteins of unknown function, as well as transposases and bacteriophage related proteins [14] and that a high proportion of them appear already as pseudogenes in *Sodalis* (unpublished results). In any case, it is clear that ISs must be involved in genomic recombination events leading to the loss of the region between two elements. Thus, similar to what is found in SOPE, ISs must be a key factor in the genome degradation that occurs in the first stages of integration to intracellular life even though the random pseudogenization of genes that have become non-essential started before their proliferation.

## 5. Concluding Remarks: Dynamics of Junk-DNA during the Evolutionary Reduction Process

Comparative studies have revealed that bacterial genomes are under selective pressures that have a deep impact on their shape and gene content, including a previously unexpected degree of diversity in gene repertoires within and between species. In addition to a set of genes that are shared by all members of a monophyletic group, there are a considerable number of genes and other functional features that can be highly specific depending on the environmental context. The evolution of these gene repertoires can be explained by processes of gene gains through HGT and duplications, and gene loses, through pseudogenization and gene excision [28]. Since it is well known that HGT has a great impact on prokaryotes [64], reductive evolutionary processes due to gene degradation and elimination must also occur very frequently in order to maintain compact genomes [31,65]. Pseudogenes must be quickly removed from the genomes, as it can be deduced from the small proportion of them those that are present simultaneously in different strains. When mutations occur in genes that are no longer needed as an adaptation to particular living conditions, the inactivated genes can remain in the genome for some time and they gradually erode in a random manner until they are completely removed [57,66], because within bacteria (as well as within several eukaryotes) there is a mutational bias toward deletions over insertions [31,67,68,69]. 

**Figure 1:**
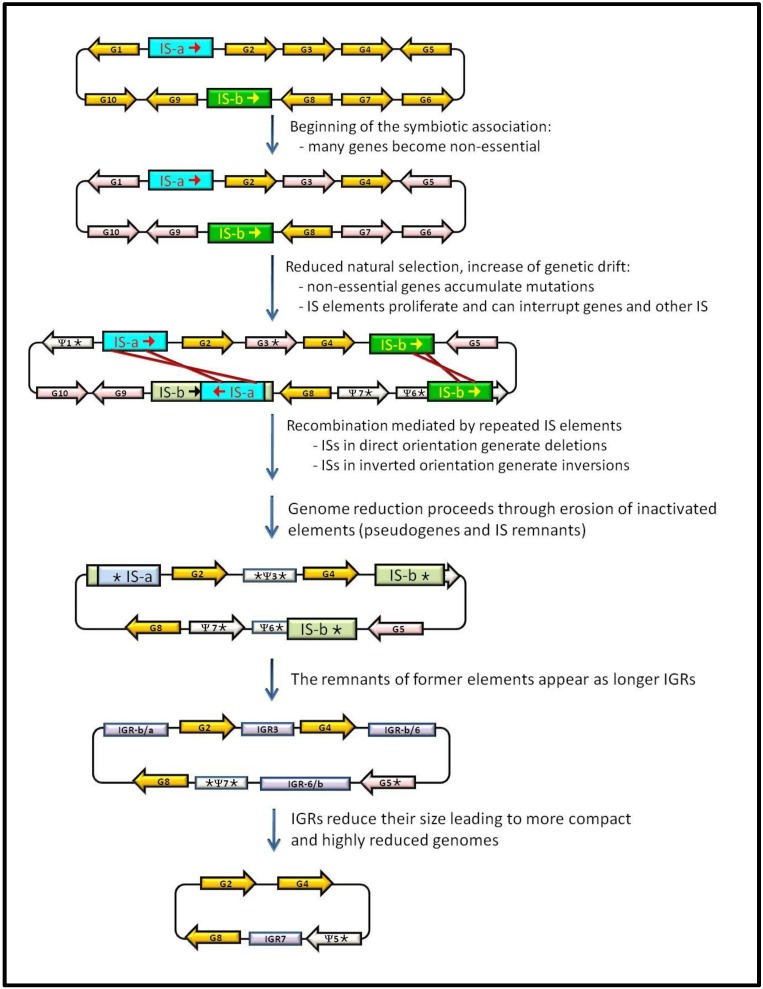
Dynamics of gain and loss of junk DNA in bacteria that establishes a host-dependent lifestyle. Details are given in the text. Orange arrows: essential genes; pink arrows: non-essential genes; white arrows: pseudogenes; blue and green boxes ISs that are active (dark colour) or inactive (light colour); light purple boxes: IGRs.

The dynamics of degradation and elimination of junk sequences has been studied in intracellular specialists by comparative genomics with their free-living relatives (Figure 1). Free-living bacteria have larger genomes with a moderate content of repeated sequences and self-propagating DNA, such as transposons, bacteriophage, a moderate amount of pseudogenes (usually between 1% and 5%) and relatively short IGRs. In the transition from free-living to host-dependent lifestyles, junk DNA increases their content, leading some times to an expansion of genome size parallel to a decrease in gene-coding capacity. The accumulation of pseudogenes in different degrees of decay (sometimes leading to long IGRs in which no traces of former pseudogenes can be detected except based on genome synteny studies), as well as the accumulation and losses of IS elements in the genomes of these bacteria can be explained by genetic and population factors. At the beginning of the association, the new rich, protected and stable niche provided by the host makes unnecessary or redundant some gene functions. Due to a decreased efficiency of the purifying selection, they can rapidly accumulate slightly deleterious mutations on genes that have become non-essential. The inefficiency of the purifying selection also explains the increase in the amount of IS elements present in these genomes at the onset of symbiosis, which in some cases seems to be accompanied by an increase in the transposition rate and the concomitant production of pseudogenes created by IS insertions. At the same time, the random genetic drift increases due to a drastic reduction of the bacterial effective population size when they are transmitted from one host to another. The uncontrolled proliferation of IS elements can lead to the loss of large stretches of genomic DNA by unequal recombination when they appear in direct orientation. This was one of the predicted effects in the model for the reductive syndrome of endosymbiont genomes, which takes place in two stages: first, a massive reduction and a second stage of pseudogenization and progressive disappearing of non-functional sequences by gene-by-gene erosion and deletion, leading to the highly compact and streamlined genomes of symbionts with a long established obligatory intracellular relationship with their hosts. IS elements also suffer the accumulation of mutations that render them inactive for transposition, thus becoming real junk DNA. The process occurs in a random manner so that some genomes can accumulate dramatic amounts of ISs (as in the case of SOPE) or lose most of them at the end of the first stage. The possibility for rapid genome degradation by means of deletion events is unlikely in the latter genomes and therefore, as it is the case of *S. symbiotica* SCc, they enter the second step of gene-by-gene degeneration while still retaining great amounts of pseudogenes which, with evolutionary time, will appear as long IGRs.
